# Bisphenol-A impairs synaptic formation and function by RGS4-mediated regulation of BDNF signaling in the cerebral cortex

**DOI:** 10.1242/dmm.049177

**Published:** 2022-07-25

**Authors:** Sung-Ae Hyun, Moon Yi Ko, Sumi Jang, Byoung-Seok Lee, Jaerang Rho, Kee K. Kim, Woo-Yang Kim, Minhan Ka

**Affiliations:** 1 Department of Advanced Toxicology Research, Korea Institute of Toxicology, KRICT, Daejeon 34114, South Korea; 2 Department of Biochemistry, Chungnam National University, Daejeon 34134, South Korea; 3 Department of Biological Sciences, Kent State University, Kent, OH 44242, USA; 4 Department of Microbiology and Molecular Biology, Chungnam National University, Daejeon 34134, South Korea

**Keywords:** Bisphenol-A, Dendritic spine, Synaptic transmission, RGS4, BDNF/NTRK2 signaling

## Abstract

Bisphenol-A (BPA) is a representative endocrine disruptor, widely used in a variety of products including plastics, medical equipment and receipts. Hence, most people are exposed to BPA via the skin, digestive system or inhalation in everyday life. Furthermore, BPA crosses the blood–brain barrier and is linked to multiple neurological dysfunctions found in neurodegenerative and neuropsychological disorders. However, the mechanisms underlying BPA-associated neurological dysfunctions remain poorly understood. Here, we report that BPA exposure alters synapse morphology and function in the cerebral cortex. Cortical pyramidal neurons treated with BPA showed reduced size and number of dendrites and spines. The density of excitatory synapses was also decreased by BPA treatment. More importantly, we found that BPA disrupted normal synaptic transmission and cognitive behavior. RGS4 and its downstream BDNF/NTRK2 pathway appeared to mediate the effect of BPA on synaptic and neurological function. Our findings provide molecular mechanistic insights into anatomical and physiological neurotoxic consequences related to a potent endocrine modifier.

## Abstract (precis)

**Summary:** Bisphenol-A (BPA) disrupts normal synaptic transmission and cognitive behavior in mice. *Rgs4* transcription factor and its downstream BDNF/NTRK2 pathway appear to mediate the effect of BPA on synaptic and neurological function.

## INTRODUCTION

Bisphenol-A (BPA), an organic synthetic chemical, is widely used in daily life, and its potential risk to humans is a major public health concern (Inadera, 2015). BPA exposure has been proposed as a risk factor for human diseases, such as cardiovascular disease, obesity, diabetes and cancers (Rochester, 2013). BPA has a high affinity to human estrogen receptors and triggers estrogenic activities (Bolli et al., 2010; Takayanagi et al., 2006). It can also disrupt androgen receptor signaling, affecting reproduction, metabolism and broad developmental processes (Delfosse et al., 2014). BPA can cross the blood–brain barrier (Hu et al., 2017; Zhou et al., 2011) and influence brain function and behavior (Perez-Lobato et al., 2016; Santoro et al., 2019). Recent studies show that prenatal BPA exposure induces childhood neuropsychiatric deficits, such as depression and anxiety, as well as social and cognition dysfunction observed in autism spectrum disorder (Miodovnik et al., 2011; Perera et al., 2016). In addition, BPA exposure is associated with neurodegeneration through oxidative stress, neural inflammation and synaptic dysfunction (Liu et al., 2016; Rezg et al., 2014; Takahashi et al., 2018). Nevertheless, there is a lack of understanding about key regulators and molecular mechanisms responsible for BPA neurotoxicity.

Neurons are polarized cells with two functionally and structurally different processes, axon and dendrites, that are required for neural transmission (Arimura and Kaibuchi, 2007). Neurons receive input signals from other neurons or glial cells through dendrites and send output messages to other cells at axon terminals (Craig and Banker, 1994; Witte and Bradke, 2008). Abnormal structures of axons and dendrites can lead to synaptic dysfunction and thereby are implicated in neurodevelopmental and neuropsychological disorder (Bellon, 2007; Kaufmann and Moser, 2000; Pardo and Eberhart, 2007). Dendritic spines are small protrusions on dendrites and form a postsynaptic compartment at excitatory synapses (Bourne and Harris, 2008). Neural activity and experience can change dendritic spine density and structure (Leuner and Gould, 2010). The dynamics of spine generation and removal in response to external stimuli are a key feature of synaptic plasticity, which is a basis of learning, memory and addiction (Engert and Bonhoeffer, 1999; Robinson and Kolb, 2004). Recent studies report that BPA suppresses neurite outgrowth and branching in PC12 neuronal cell lines and human neural progenitor cells (Habauzit et al., 2014; Seki et al., 2011; Wu et al., 2016). BPA exposure reduces dendritic spines as well (Eilam-Stock et al., 2012; Hu et al., 2017). Functionally, maternal BPA exposure has been shown to impair recognition memory via inhibition of the brain-derived neurotrophic factor (BDNF) pathway (Wang et al., 2016). BDNF and its receptor, neurotrophic tyrosine kinase receptor type 2 [NTRK2; also known as tropomyosin receptor kinase B (TRKB)] play an important role in differentiation, maturation and survival of neurons (Acheson et al., 1995; Warita et al., 2013). In particular, synaptic maturation and transmission require BDNF signaling (Kellner et al., 2014; Zagrebelsky and Korte, 2014). BDNF signaling activates the transcription factors CREB and CREB-binding protein (CBP; also known as CREBBP) related to gene expression for neural plasticity (Finkbeiner et al., 1997; Kaplan and Miller, 1997; Mattson et al., 2004). However, the molecular mechanisms underlying the detrimental effects of BPA on neurite formation and synaptic function are unclear.

Regulator of G protein signaling 4 (RGS4) belongs to the R4/B subfamily and is highly expressed throughout the brain including the prefrontal cortex (PFC), hippocampus and striatum (Heraud-Farlow et al., 2013; Larminie et al., 2004; Paspalas et al., 2009). Additionally, RGS4 plays an essential role in synaptic signaling and plasticity by regulation of G protein-coupled receptors (GPCRs) such as dopaminergic, serotonergic, noradrenergic, glutamatergic or opioid receptors (Gerber et al., 2016). Microarray and genomic analysis studies show that transcript level of *RGS4* in the PFC are decreased in a diagnosis-specific manner in schizophrenia patients (Chowdari et al., 2002; Mirnics et al., 2001). In this study, we reveal that BPA regulates neurite formation and synaptic function in mouse cortical pyramidal neurons. BPA exposure resulted in aberrant dendrite branching, spine formation and synaptic transmission. These morphological and physiological alterations highly correlated with cognitive deficits in the mice. BPA exposure downregulated RGS4 levels and subsequently reduced RGS4-mediated BDNF expression. Our data suggest that the RGS4–BDNF pathway may serve as a regulatory mechanism for BPA-induced synaptic and cognitive dysfunction in mice.

## RESULTS

### BPA exposure alters expression of synapse-related genes in cortical neurons

**Fig. 1.**
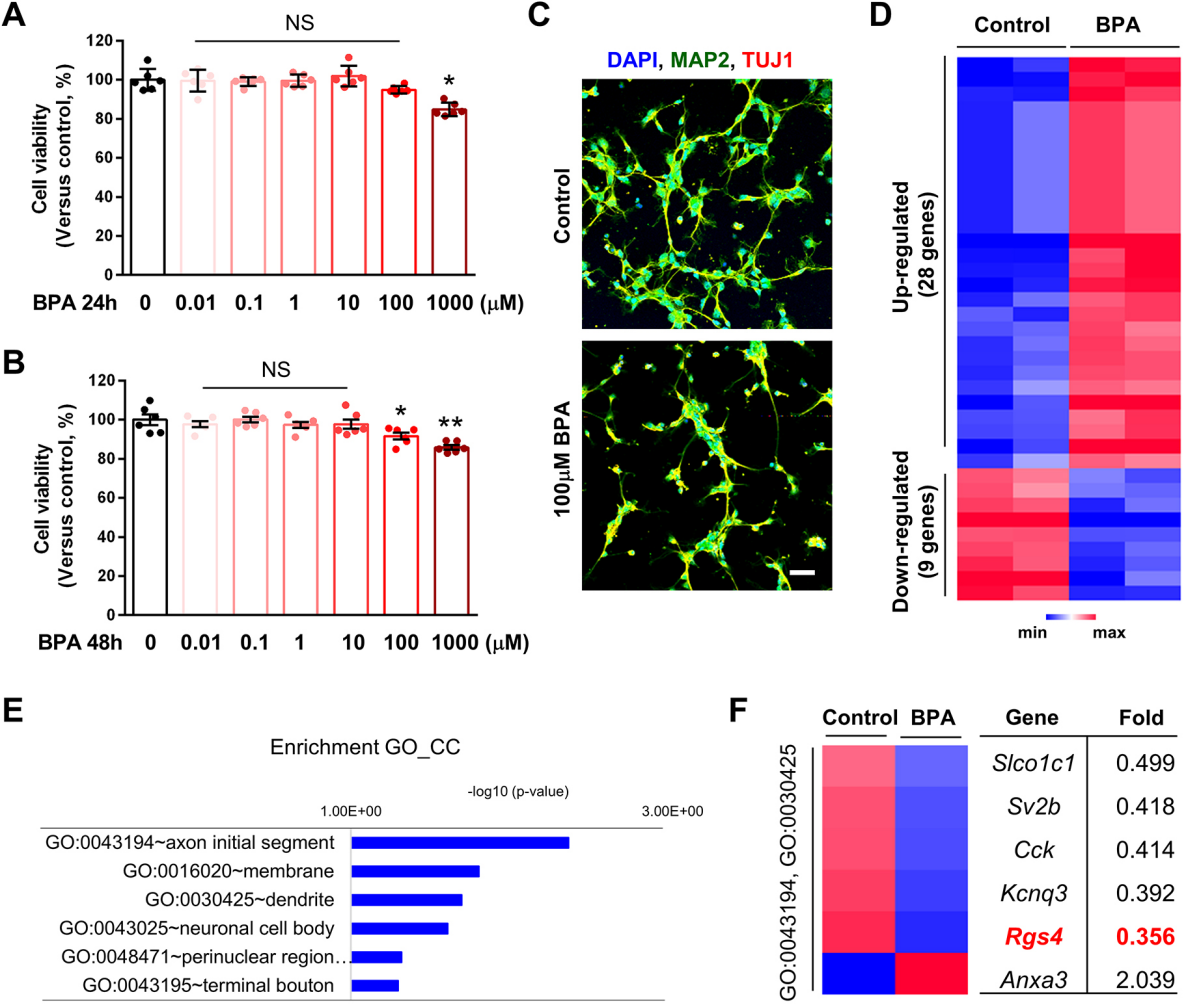
**Gene expression changes in cultured cortical neurons after bisphenol-A (BPA) exposure.** (A,B) Cell viability: WST-8 assay. Histograms represent the percentage, with respect to control cells, of viable cells after exposure to 0.01, 0.1, 1, 10, 100 or 1000 μM BPA for 24 h or 48 h. Statistical significance was determined by two-way ANOVA with Bonferroni correction. Data are shown as relative changes versus controls. NS, not significant; *P<0.05, **P<0.01. (C) BPA suppressed neurite outgrowth in cultured cortical neurons. Cultured cortical neurons were treated with 0.1% DMSO or BPA (100 μM) for 24 h. Neurites were assessed by immunostaining using anti-MAP2 and anti-TUJ1 antibodies. Scale bar: 10 μm. (D) BPA exposure changes the levels of mRNAs in the cortical neurons. Heatmap of RNA-sequencing transcriptome analysis for different genes in brains of control or BPA-exposed groups (fold change >2; *P*<0.05). (E) The significantly enriched GO_CC terms for upregulated genes by BPA. (F) Heatmap of differentially expressed genes associated with enriched GO_CC terms [axon initial segment (GO: 0043194): *Kcnq3*, *Cck*; neuronal cell body (GO:0030425): *Kcnq3*, *Cck*, *Anxa3*] and *Rgs4*.

To examine the cytotoxic effect of BPA in neural cells, we cultured cortical neurons from embryonic day (E)15 mice for 3 days and treated them with varying doses of BPA (0.01-1000 µM) for 24 h or 48 h. Cell survival was assessed by WST-8 assay. BPA induced cytotoxicity in a concentration-dependent manner (Fig. 1). Lower concentrations (0.01-100 µM) of BPA for either 24 h (Fig. 1A) or 48 h (Fig. 1B) did not significantly change cell viability, whereas higher doses (1000 µM) decreased cell survival. Based on the dose-responsive effect, we chose the 100 µM BPA concentration to perform transcriptomic analysis. Cortical neurons were exposed to either BPA (100 µM) or 0.1% dimethyl sulfoxide (DMSO; control) for 24 h (Fig. 1C), and the global transcriptome pattern was profiled using microarray. First, we found 269 differentially expressed mRNAs, of which 158 were upregulated and 111 were downregulated. We found that 37 mRNAs were statistically significantly changed (fold change >2; *P*<0.05). Among these, 28 mRNAs were upregulated and nine mRNAs were downregulated in BPA-exposed neurons compared with controls (Fig. 1D). Gene Ontology (GO) analyses were performed to explore the potential areas and functions of differentially expressed mRNAs. The axon initial segment and dendrites were the most targeted regions (Fig. 1E). We narrowed down to five downregulated genes: *Slco1c1*, *Sv2b*, *Cck*, *Kcnq3* and *Rgs4*. All these genes are known to regulate neurite formation and function in neurons (Fig. 1F). These results suggest that BPA exposure triggers abnormal neural function in cortical pyramidal neurons.

### BPA impairs neurite outgrowth and branching in cortical neurons

**Fig. 2.**
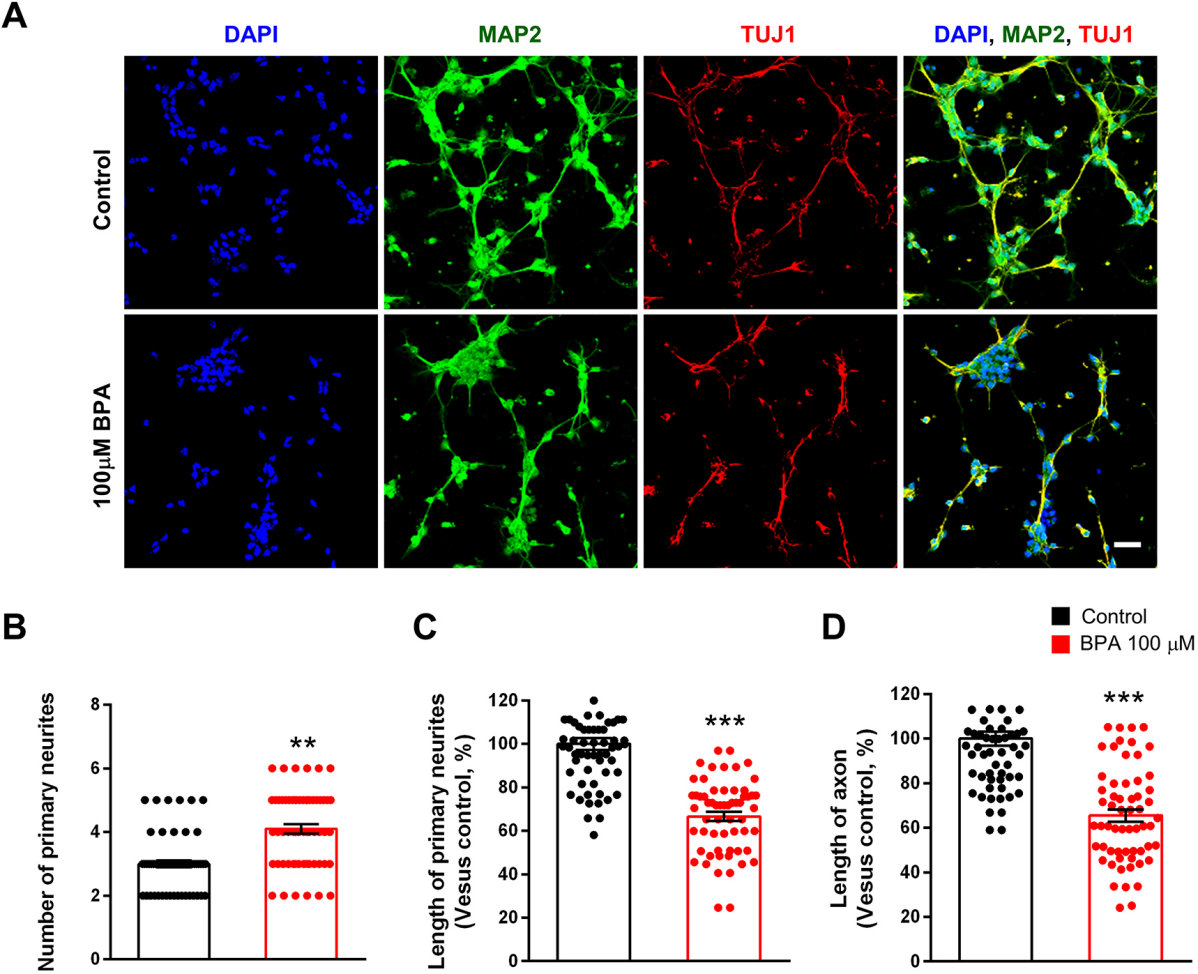
**Abnormal neurite architecture in cultured cortical neurons after BPA exposure.** (A) BPA initially suppressed neurite outgrowth in cultured cortical neurons. At 5 days *in vitro* (DIV5), cultured cortical neurons were treated with saline or BPA (100 μM) for 24 h. Neurites were assessed by immunostaining using an anti-MAP2 antibody. Axons were assessed by immunostaining using an anti-TUJ1 antibody. Scale bar: 10 μm. (B) Quantification of the number of primary neurites in each condition. *n*=60 neurons for control; *n*=60 neurons for the BPA-exposed condition. (C) Quantification of the length of primary neurites in each condition. *n*=60 neurons for control; *n*=60 neurons for the BPA-exposed condition. (D) Quantification of the length of axons in each condition. *n*=60 neurons for control; *n*=60 neurons for the BPA condition. Statistical significance was determined by one-way ANOVA with Bonferroni correction. Data are shown as relative changes versus controls. **P<0.01, ***P<0.001.

We assessed the BPA effect on neurite elongation and branching. We cultured cortical pyramidal neurons from E15 mice and treated these cells with BPA (100 μM) or 0.1% DMSO for 3 days, followed by immunostaining with anti-microtubule-associated protein 2 (MAP2) and neuron-specific anti-class III beta-tubulin [TUJ1 (also known as TUBB3) clone] antibodies. The number of MAP2-stained dendrites was increased by 29% in BPA-exposed neurons compared with controls (Fig. 2A,B). However, the length of dendrites was decreased by 30% in the BPA-exposed condition (Fig. 2A,C). The length of TUJ1-stained axons was also decreased by 40% in BPA-exposed neurons compared with control neurons (Fig. 2A,D). These results show that BPA exposure leads to abnormal neurite morphology in cortical pyramidal neurons.

### Dendritic spine deficits in BPA-treated cortical neurons

**Fig. 3.**
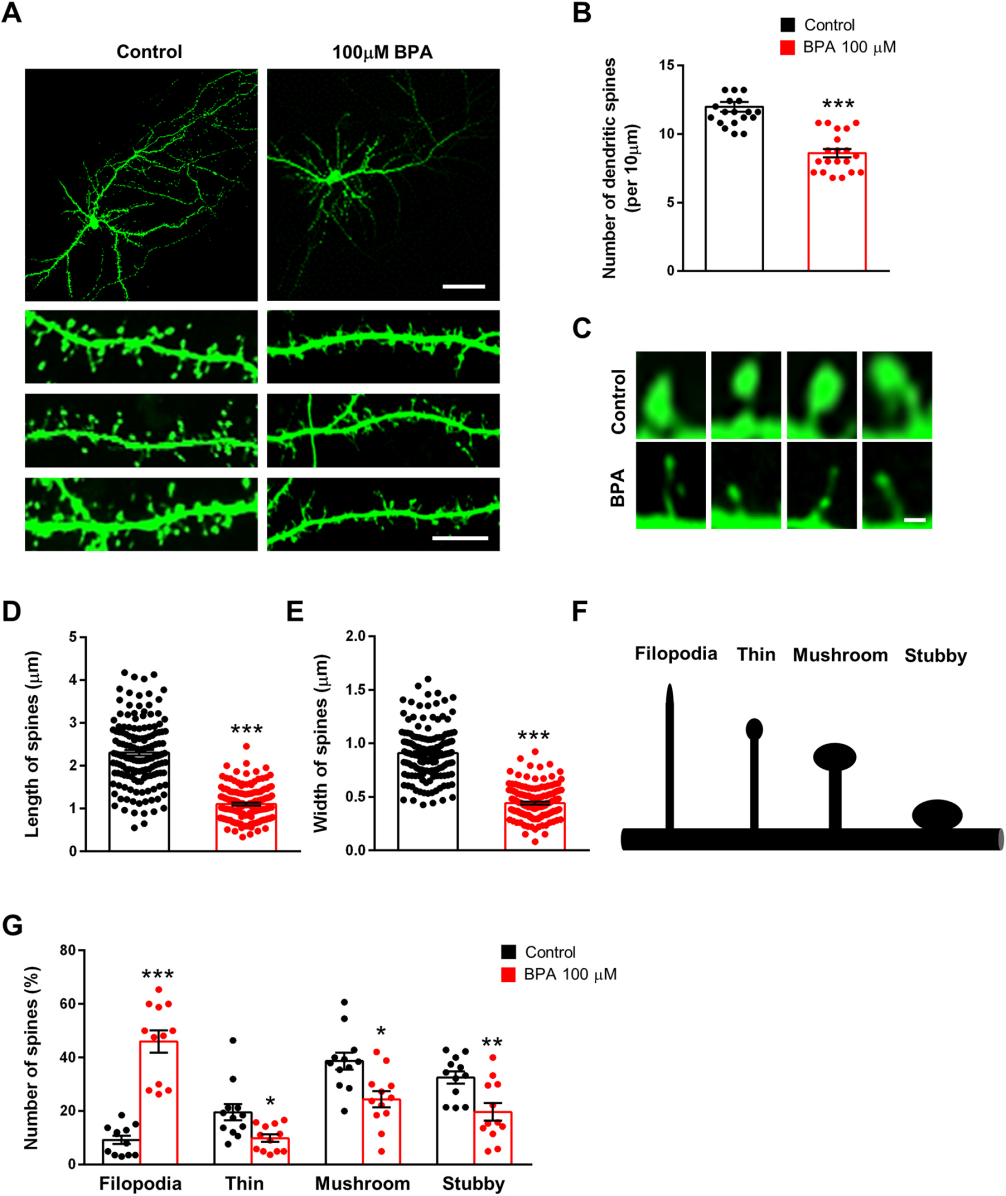
**BPA regulates dendritic spines in cultured cortical neurons.** (A) BPA modifies dendritic spine density in cultured cortical neurons. Cultured cortical neurons were isolated from E15 embryos, cultured and transfected with a GFP plasmid at DIV10. After 4 days, neurons were treated with 100 μM BPA for 24 h. Scale bars: 25 μm (top panels) and 10 μm (bottom panels). (B) Quantification of the number of dendritic spines in each condition. *n*=20 neurons from three independent cultures using three mice for each condition. Statistical significance was determined by one-way ANOVA with Bonferroni correction. Data are shown as relative changes versus controls. ***P<0.001. (C) Higher-magnification images of dendritic spines. Exposure of BPA leads to abnormal dendritic spine morphology. Scale bar: 0.5 μm. (D,E) Quantification of the width and length of dendritic spines in each condition. *n*=150 spines for control; *n*=150 spines for the BPA-exposed condition. Statistical significance was determined by one-way ANOVA with Bonferroni correction. Data are shown as relative changes versus controls. ***P<0.001. (F) Different types of dendritic spines (mushroom, thin, stubby and filopodia). (G) BPA remodels the composition of dendritic spine types in cultured cortical neurons. *n*=12 cultured cortical neurons and 300 dendritic spines for each condition. Statistical significance was determined by two-way ANOVA with Bonferroni correction. Data are shown as relative changes versus controls. *P<0.05, **P<0.01, ***P<0.001.

The dendritic spine is a major structure that receives synaptic input (Chen and Hsueh, 2012; Spacek and Harris, 1998). Thus, we sought to determine whether BPA also alters dendritic spines. We cultured cortical neurons from E15 mice for 10 days and transfected them with a green fluorescent protein (GFP) plasmid. After 4 days, cortical neurons were exposed to either BPA or 0.1% DMSO for 24 h, and dendritic spines were assessed by GFP immunostaining. Neurons exposed to BPA displayed a 30% decrease in the number of spines compared with control neurons (Fig. 3A,B). We also assessed the number of dendritic spines in hippocampal neurons. Consistently, hippocampal neurons exposed to BPA displayed a 23% decrease in the number of spines compared with controls (Fig. S1A,B). BPA-exposed spines appeared shorter than control spines, with smaller heads and narrower necks. The length of dendritic spines was decreased by 52% in the BPA-exposed condition (Fig. 3C,D), and the size of spine heads was reduced by 51% as well (Fig. 3C,E). Dendritic spine morphology is important for normal synaptic function (Lamprecht and LeDoux, 2004). Dendritic spines can be classified into four different categories – such as filopodia, thin, mushroom or stubby spines – by morphology (Ka and Kim, 2018). Filopodia are typically longer (>2 μm) without a clear head; thin spines have a thin and long neck (>1 μm) and a small head; mushroom spines have a short and narrow neck (<1 μm) and a large head (>0.6 μm); and stubby spines have a head but no neck (Fig. 3F). We found that BPA exposure induced abnormal spine morphology. There was a 400% increase in filopodia-like spines in BPA-exposed neurons compared with controls. Furthermore, the numbers of thin, mushroom and stubby spines were decreased by 50%, 37% and 39%, respectively, in BPA-exposed neurons (Fig. 3G). Together, these results show that BPA exposure leads to dendritic spine malformation in cortical neurons.

### Synapse formation and function are disrupted by BPA exposure

**Fig. 4.**
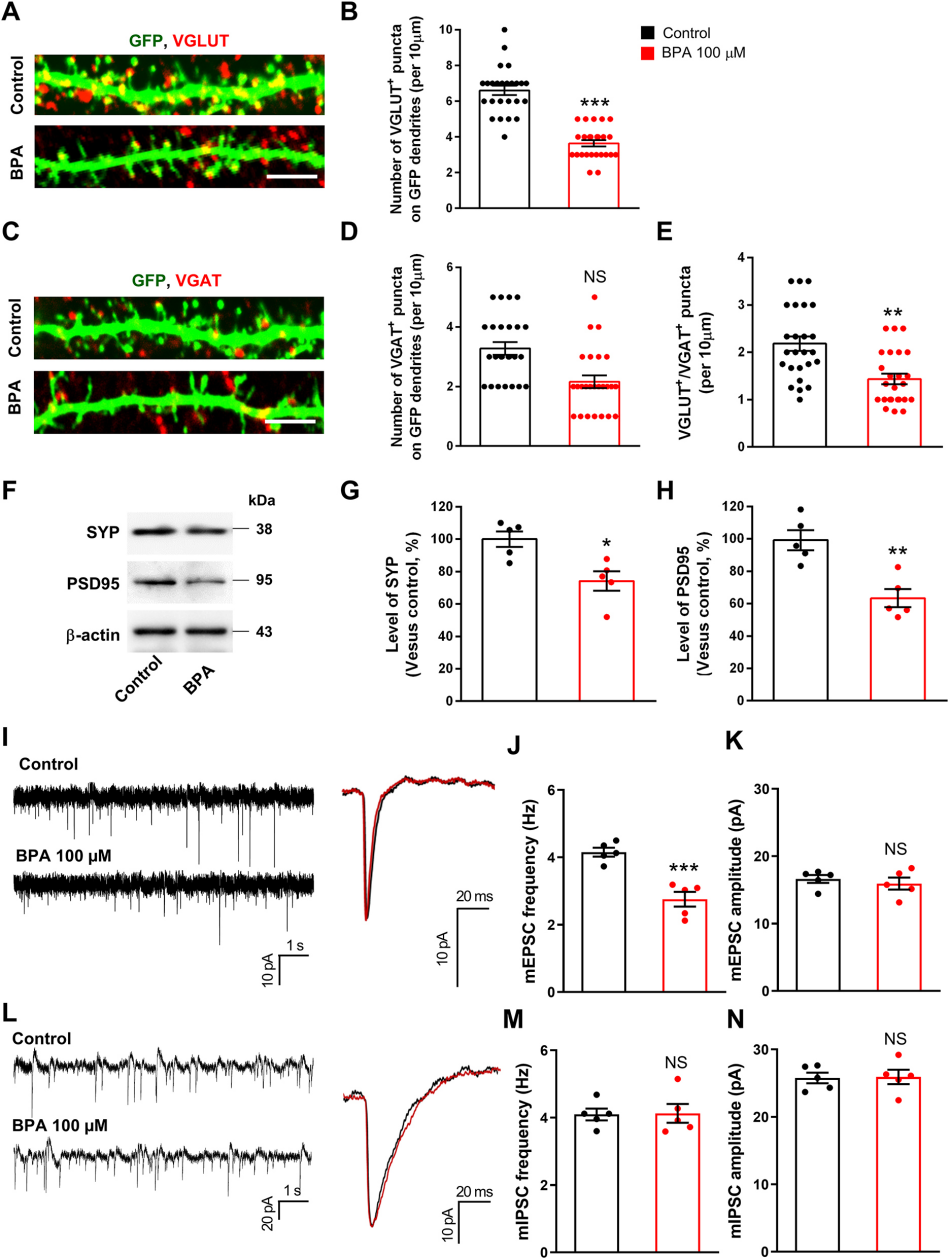
**BPA affects synaptic formation in cultured cortical neurons.** (A) Cultured cortical neurons from E15 mice were cultured for 10 days and transfected with a GFP plasmid. After 4 days, neurons were treated with 100 μM BPA for 24 h. Excitatory synapses were assessed by immunostaining using an anti-VGLUT antibody. Scale bar: 10 μm. (B) Quantification of the number of excitatory synapses shown in A. *n*=25 neurons from three independent cultures using three mice for each condition. Statistical significance was determined by one-way ANOVA with Bonferroni correction. Data are shown as relative changes versus controls. ***P<0.001. (C) BPA induces no changes in the number of inhibitory synapses in cultured cortical neurons. Inhibitory synapses were assessed by immunostaining using an anti-VGAT antibody. Scale bar: 10 μm. (D) Quantification of inhibitory synapse numbers shown in C. *n*=25 neurons from three independent cultures using three mice for each condition. Statistical significance was determined by one-way ANOVA with Bonferroni correction. Data are shown as relative changes versus controls. (E) BPA changes the balance of excitatory and inhibitory synaptic puncta. *n*=25 neurons from three independent cultures using three mice for each condition. Statistical significance was determined by one-way ANOVA with Bonferroni correction. Data are shown as relative changes versus controls. **P<0.01. (F) BPA decreases presynaptic and postsynaptic molecules in cultured cortical neurons. Cellular lysates were isolated from neurons treated with 100 μM BPA for 24 h. Western blotting was performed with anti-SYP and anti-PSD95 antibodies. (G,H) Quantification of protein levels shown in F. The levels of protein were normalized to β-actin expression. *n*=5 independent cultures using five mice. Statistical significance was determined by one-way ANOVA with Bonferroni correction. Data are shown as relative changes versus controls. *P<0.05, **P<0.01. (I) Representative whole-cell voltage-clamp recording showing miniature excitatory postsynaptic currents (mEPSCs) from control and BPA-exposed neurons. (J,K) Quantification of the frequency and amplitude of mEPSCs shown in I. Statistical significance was determined by one-way ANOVA with Bonferroni correction. Data are shown as relative changes versus controls. ***P<0.001. (L) Representative whole-cell voltage-clamp recording showing mIPSCs in control and BPA-exposed neurons. (M,N). Quantification of the frequency and amplitude of mEPSCs shown in L. Statistical significance was determined by one-way ANOVA with Bonferroni correction. Data are shown as relative changes versus controls. NS, not significant.

We examined synapses in cortical neurons treated with BPA. Synapses on cultured neurons were immunostained with antibodies specific to synaptic markers, vesicular glutamate transporter (VGLUT; also known as SLC17A; excitatory synapses) and vesicular GABA transporter (VGAT; also known as SLC32A1; inhibitory synapses). We found that the number of excitatory synapses was decreased by 42% in BPA-exposed neurons compared with control neurons (Fig. 4A,B). Interestingly, the number of inhibitory synapses was not changed following BPA exposure (Fig. 4C,D). We also assessed synapses in hippocampal neurons. Consistently, we found that the number of excitatory synapses was decreased by 35% in BPA-exposed hippocampal neurons; however, the number of inhibitory synapses was not changed following BPA exposure (Fig. S1C-F). Balanced transmission of excitatory and inhibitory synapses is critical for normal brain function. We found that the ratio of VGLUT to VGAT puncta was decreased in BPA-exposed neurons (Fig. 4A,C,E). We determined the levels of excitatory synaptic markers, such as synaptophysin (SYP; presynaptic marker) and postsynaptic density protein 95 (PSD95; also known as DLG4; postsynaptic marker) in cortical lysates following BPA exposure. We found that the level of SYP was decreased by 26% in BPA neurons compared with controls (Fig. 4F,G). Likewise, the level of PSD95 was decreased by 37% in BPA-exposed neurons (Fig. 4F,H).

Next, we sought to determine the functional relevance of abnormal synaptic morphology in BPA-exposed neurons. Thus, we investigated whether BPA exposure alters excitatory and inhibitory synaptic transmission by whole-cell voltage-clamp recordings. The frequency of miniature excitatory postsynaptic currents (mEPSCs) was decreased in BPA-exposed neurons compared with control neurons (Fig. 4I,J). The amplitude of mEPSCs was not significantly changed (Fig. 4I,K). Interestingly, BPA treatment led to no change in the frequency and amplitude of miniature inhibitory postsynaptic currents (mIPSCs) (Fig. 4L-N).

**Fig. 5.**
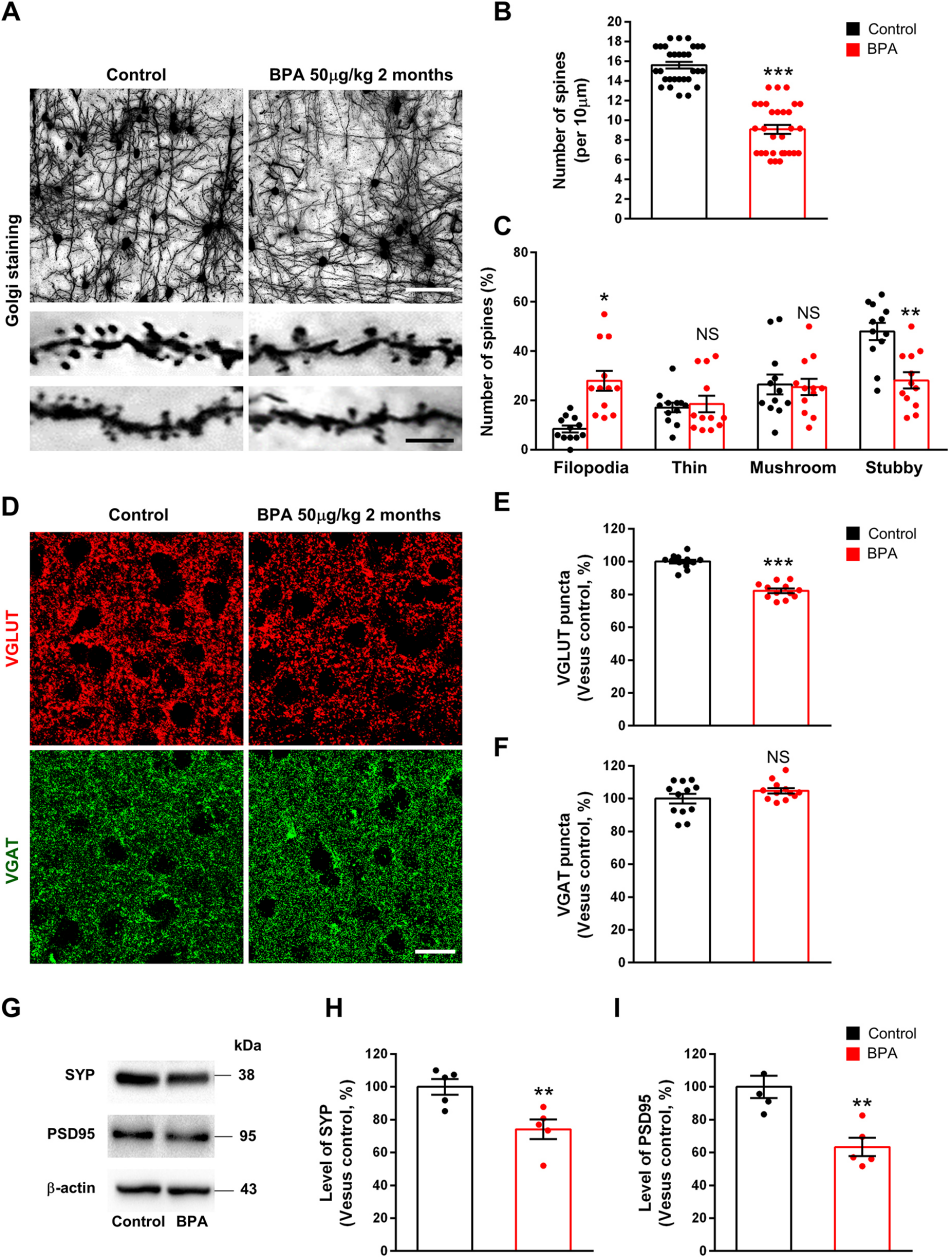
**BPA administration changes dendritic spines and decreases excitatory synapses in the cerebral cortex.** (A) Dendritic spines were assessed in cortical tissues from mice exposed to either BPA (50 µg/kg) or saline (control) orally once a day for 2 months. Golgi staining showed a decrease in dendritic spines in BPA-exposed cortices. Scale bars: 50 μm (top panels) and 10 μm (bottom panels). (B) Quantification of the number of dendritic spines in each condition. *n*=30 neurons from three mice for each condition. Statistical significance was determined by two-way ANOVA with Bonferroni correction. Data are shown as relative changes versus controls. ***P<0.001. (C) BPA remodels the composition of dendritic spine types in cerebral cortices. *n*=12 sections from three mice for each condition. Statistical significance was determined by two-way ANOVA with Bonferroni correction. Data are shown as relative changes versus controls. *P<0.05, **P<0.01. (D) Mice were administered either BPA (50 µg/kg) or saline (control) orally once a day for 2 months. Then, excitatory and inhibitory synapses were assessed by immunostaining with anti-VGLUT and anti-VGAT antibodies. Scale bar: 20 μm. (E,F) Quantification of the number of synaptic puncta in each condition. *n*=12 sections from three mice for each condition. Statistical significance was determined by two-way ANOVA with Bonferroni correction. Data are shown as relative changes versus controls. ***P<0.001. (G) The levels of synaptic proteins were decreased by BPA administration in the cerebral cortices. After BPA exposure, western blotting was performed using anti-SYP and anti-PSD95 antibodies. (H,I) Quantification of the protein levels shown in G. The levels of protein were normalized to β-actin expression. *n*=5 sections using five mice. Statistical significance was determined by one-way ANOVA with Bonferroni correction. Data are shown as relative changes versus controls. **P<0.01. NS, not significant.

Finally, we examined the effect of BPA on dendritic spines and synapses *in vivo*. Three-month-old male mice were administered either BPA (50 µg/kg) or 0.1% DMSO (control) orally once a day for 2 months. Brains were isolated within 1 h of the last BPA administration and subjected to Golgi staining. We found that the number of dendritic spines was decreased by 42% in BPA-exposed cortices compared with controls (Fig. 5A,B). Similar to the findings in cultured neurons, BPA brains displayed abnormal spines. The number of filopodia-like spines was increased by 270% in BPA-exposed cortices compared with controls, whereas the number of stubby spines was decreased by 41% in BPA-exposed cortices compared with controls (Fig. 5A,C). We also assessed excitatory and inhibitory synapses *in vivo*. The number of VGLUT puncta was decreased by 18% in BPA-exposed cortices compared with control cortices (Fig. 5D,E). However, BPA administration did not alter the number of VGAT puncta in cortices (Fig. 5D,F). The levels of SYP and PSD95 measured by western blotting were decreased by 30% and 21%, respectively, in BPA-treated mice (Fig. 5G-I). These results confirm the *in vitro* findings on BPA effects on synapse formation. Altogether, our data show that BPA exposure causes alteration to synaptic morphology and function in cortical neurons.

### BPA suppresses BDNF/NTRK2 signaling through *Rgs4* downregulation

**Fig. 6.**
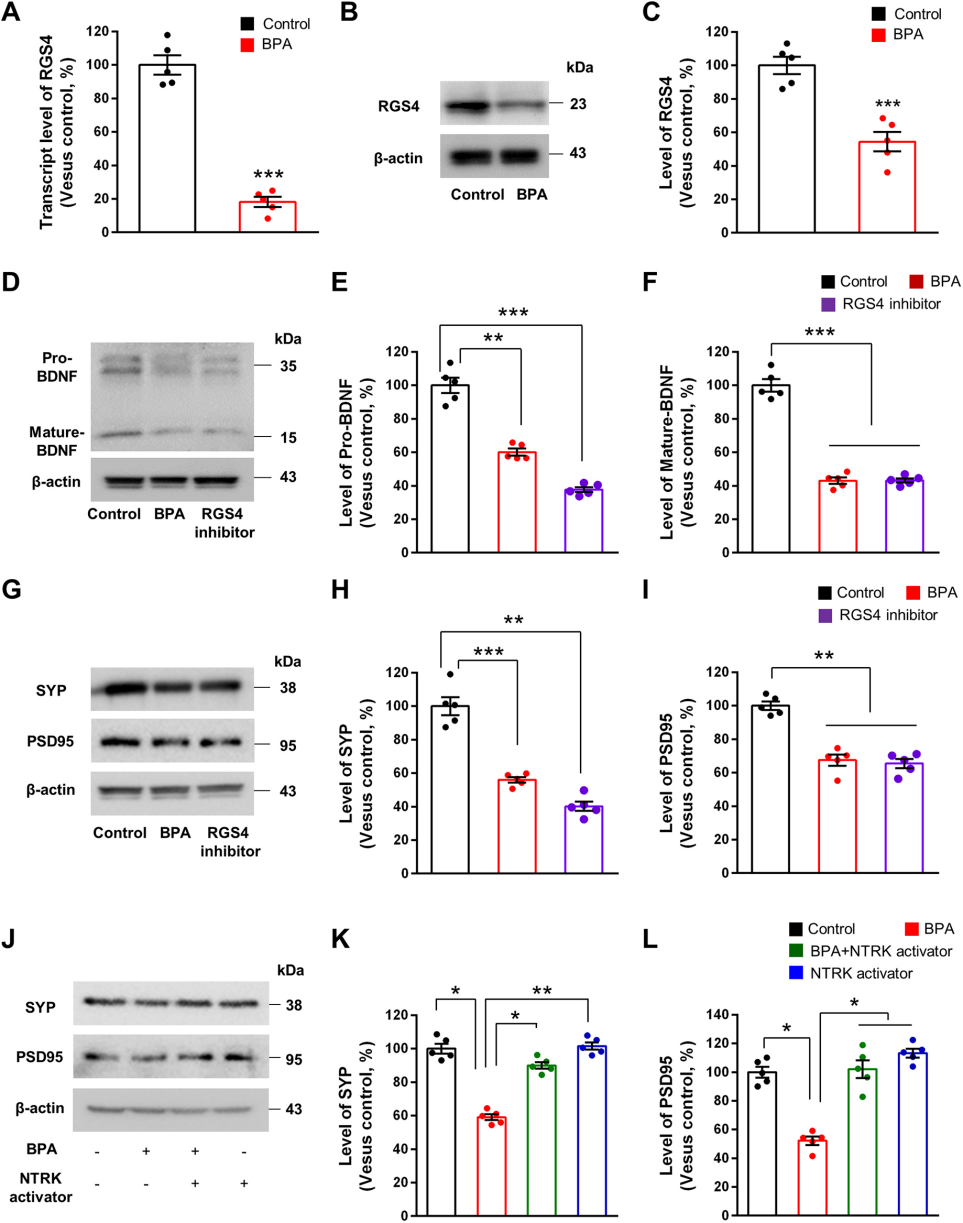
**BPA inactivates BDNF/NTRK2 signaling by regulating *Rgs4* transcription.** (A) *Rgs4* mRNA levels were assessed by RT-PCR in cultured cortical neurons treated with 100 μM BPA for 24 h. Quantification of the transcript level of *Rgs4* in each condition. *n*=5 times for each condition. Statistical significance was determined by one-way ANOVA with Bonferroni correction. Data are shown as relative changes versus controls. ***P<0.001. (B) BPA decreases RGS4 expression in cultured cortical neurons. Cellular lysates were isolated from neurons treated with 100 μM BPA for 24 h. Western blotting was performed with an anti-RSG4 antibody. (C) Quantification of RGS4 levels shown in B. The levels of RGS4 were normalized to β-actin expression. *n*=5 independent cultures using five mice. Statistical significance was determined by one-way ANOVA with Bonferroni correction. Data are shown as relative changes versus controls. ***P<0.001. (D) RGS4 inhibitor, CCG50014, decreases BDNF expression in cultured cortical neurons. Cellular lysates were isolated from neurons treated with 10 μM CCG50014 or 100 μM BPA for 24 h. Western blotting was performed with an anti-BDNF antibody. (E,F) Quantification of the protein levels shown in D. The levels of protein were normalized to β-actin expression. *n*=5 independent cultures using five mice. Statistical significance was determined by one-way ANOVA with Bonferroni correction test. Data are shown as relative changes versus controls. **P<0.01, ***P<0.001. (G) RGS4 inhibitor decreases presynaptic and postsynaptic molecules in cultured cortical neurons. Western blotting was performed with anti-SYP and anti-PSD95 antibodies. (H,I) Quantification of protein levels shown in G. The levels of protein were normalized to β-actin expression. *n*=5 independent cultures using five mice. Statistical significance was determined by one-way ANOVA with Bonferroni correction. Data are shown as relative changes versus controls. **P<0.01, ***P<0.001. (J) NTRK activator, 7,8-DHF, restores presynaptic and postsynaptic molecules in BPA-exposed cortical neurons. Cellular lysates were isolated from neurons treated with 100 μM BPA or 100 μM BPA+500 μM 7,8-DHF for 24 h. Western blotting was performed with anti-SYP and anti-PSD95 antibodies. (K,L) Quantification of the protein levels shown in J. The levels of protein were normalized to β-actin expression. *n*=5 independent cultures using 5 mice. Statistical significance was determined by one-way ANOVA with Bonferroni correction. Data are shown as relative changes versus controls. *P<0.05, **P<0.01.

We confirmed the microarray results on *Rgs4* through RT-PCR and western blotting. Total RNAs were isolated from neuronal cultures treated with BPA for 24 h. RT-PCR showed that the *Rgs4* mRNA level was reduced by 82% in BPA-exposed neurons, compared with the level in control neurons (Fig. 6A). Western blotting also revealed 43% decreased expression of RGS4 in BPA-exposed neurons compared with control neurons (Fig. 6B,C).

We investigated whether RGS4 regulates expression of BDNF and synaptic proteins in cortical pyramidal neurons. We first measured the BDNF levels in cortical neurons treated with an RGS4 inhibitor, CCG50014. We found that RGS4 inhibition reduced the levels of pro-BDNF and mature-BDNF by 62% and 57%, respectively, compared with the control condition (Fig. 6D-F). The extent of BDNF reduction was comparable to that after BPA exposure as there was no significant difference between BPA- and CCG50014-exposed conditions. Next, we assessed the number of dendritic spines in cortical neurons after CCG50014 treatment. Cortical neurons exposed to CCG50014 displayed a 22% decrease in the number of spines compared with controls (Fig. S2A,B). We also examined the levels of excitatory synaptic markers SYP and PSD95 in cultured neurons after CCG50014 treatment. The levels of SYP and PSD95 were decreased by 60% and 35%, respectively, in CCG50014-treated neurons compared with control neurons (Fig. 6G-I). Finally, we examined whether activation of BDNF/NTRK2 signaling rescues SYP and PSD95 expression in cortical neurons treated with BPA. Cortical neurons were co-treated with an NTRK activator, 7,8-dihydroxyflavone (7,8-DHF), and BPA for 24 h. Western blotting showed that the levels of SYP and PSD95 were decreased by 40% and 46%, respectively, in BPA-exposed neurons (Fig. 6J-L). However, co-treatment with 7,8-DHF reversed the inhibitory effect of BPA on SYP and PSD95 expression (Fig. 6J-L). These results suggest that BPA suppresses synapse gene expression by downregulating *Rgs4* and BDNF signaling.

### BPA exposure results in abnormal mouse behavior

**Fig. 7.**
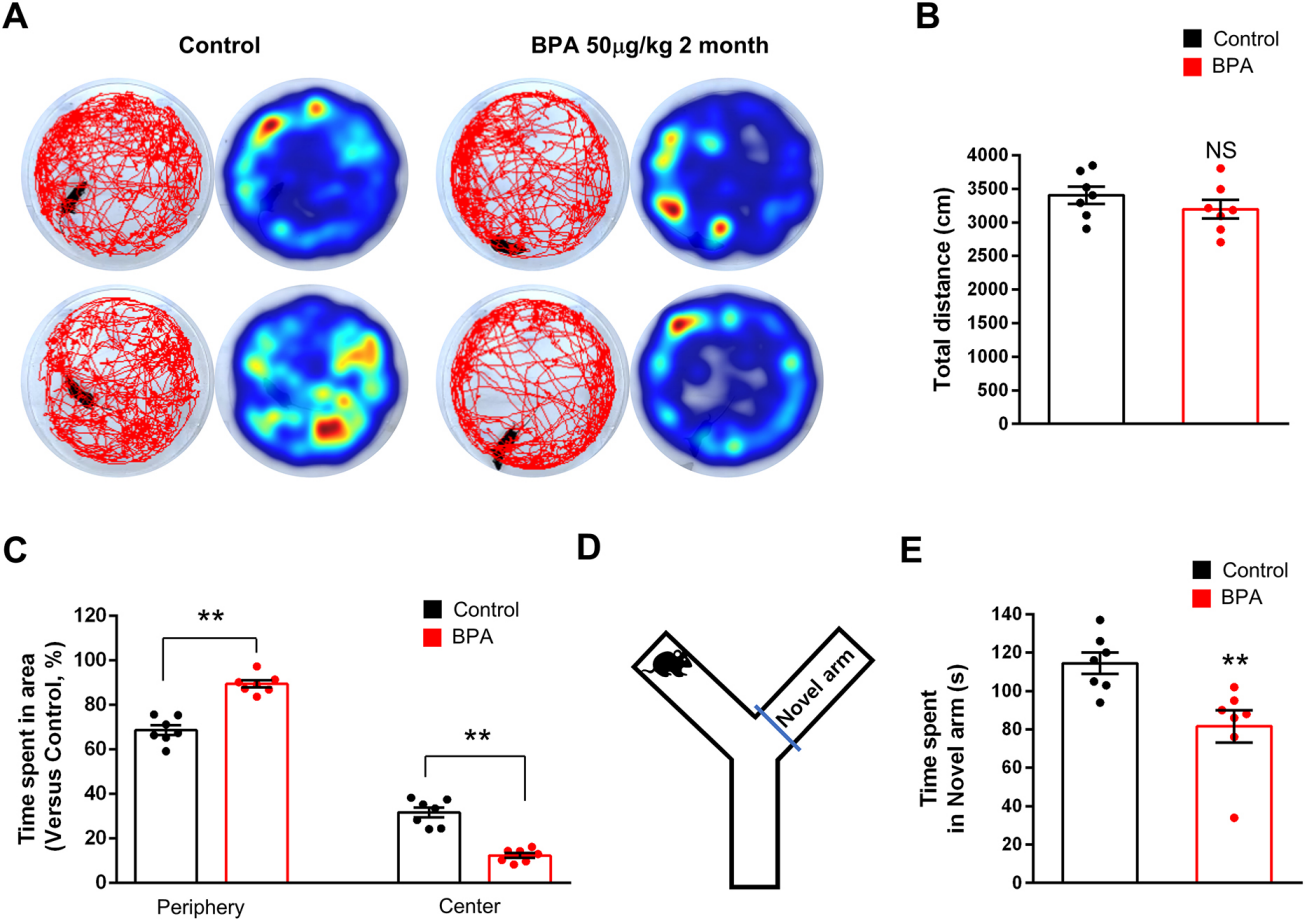
**BPA administration results in abnormal mouse behaviors.** (A) Representative images of motor activity in control and BPA-exposed mice at 2 months. (B,C) Motor activities were assessed by measuring the total distance of movement during the open field test. Total time spent in the center and periphery were quantified in the open field test. *n=*7 mice for each condition. Statistical significance was determined by one-way ANOVA with Bonferroni correction. Data are shown as relative changes versus controls. NS, not significant; **P<0.01. (D) Representative image of task for E. (E) Total time spent in the novel arm was quantified in the Y-maze test. *n=*7 mice for each condition. Statistical significance was determined by one-way ANOVA with Bonferroni correction. Data are shown as relative changes versus controls. **P<0.01.

We assessed anxiety and cognitive behaviors in mice exposed to BPA. Three-month-old male mice were treated with either BPA (50 µg/kg) or 0.1% DMSO (control) orally once a day for 2 months. First, we performed the open field test. We found no significant difference in the total distance traveled between BPA-exposed and control mice (Fig. 7A,B). However, BPA mice showed elevated anxiety behavior, spending significantly less time in the center of the field and attempting fewer center entries (Fig. 7A,C). The time spent in the center area was decreased by 53% in BPA-treated mice compared with controls. These results show that BPA exposure induces heightened anxiety-like behavior. Next, using the Y-maze test, we assessed spatial learning and memory. We found that BPA-exposed mice spent 42% less time in the novel arm than control mice (Fig. 7D,E). These results show that BPA exposure disrupts memory processing in mice.

## DISCUSSION

**Fig. 8.**
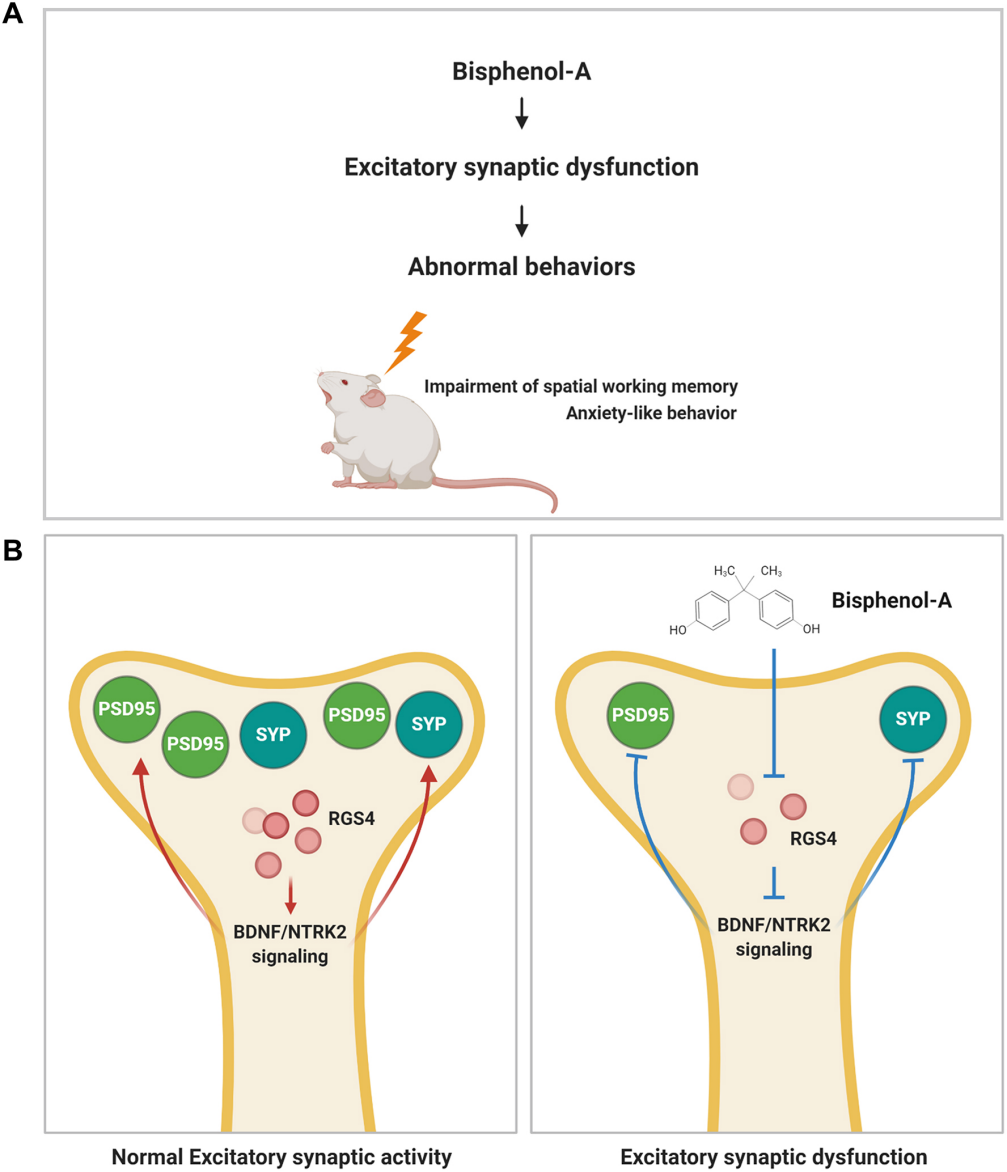
**Schematic illustrating the effect of BPA on synaptic formation and function.** (A,B) BPA inactivates *Rgs4* transcription, leading to the downregulation of BDNF/NTRK2 signaling in cortical neurons. Inactivation of BDNF/NTRK2 signaling induces abnormal synaptic architecture and behaviors.

In this study, we provide evidence that BPA alters dendritic spine formation and excitatory synaptic function in cortical pyramidal neurons. Dysregulation of RGS4 and BDNF signaling is associated with BPA-induced abnormal spine and synapse function (Fig. 8). Our results provide novel insights into anatomical and molecular targets for BPA-mediated consequences of synaptic malformation and dysfunction, and could have implications for therapeutic development for neurotoxin-mediated endocrine disruption.

We found that BPA administration altered dendritic spine density and morphology, resulting in synaptic dysfunction and abnormal mouse behaviors (Della Seta et al., 2006; Leranth et al., 2008; Tian et al., 2010). Prenatal exposure to BPA induces abnormal abnormalities in fetal hippocampus and reduces hippocampal spine synapses in non-human primates (Elsworth et al., 2013). In our study, BPA exposure increased filopodia-like spines and decreased thin, mushroom and stubby spines in cortical pyramidal neurons. BPA thus controls neural activity and behaviors by modifying synaptic connections. It has been reported that BPA impairs memory and reduces dendritic spine density in hippocampus (Eilam-Stock et al., 2012). Dendritic spines are major substrates of excitatory synapses wherein glutamate receptors, such as the NMDA and AMPA receptors, are essential components of postsynaptic activity (Hanley, 2008; Petralia et al., 1994). Moreover, BPA exposure decreases glutamate receptor expression and synaptic transmission (Hu et al., 2017). Consistently, we have shown that BPA decreases the number of excitatory synapses in cerebral cortex. Interestingly, BPA exposure did not alter the density of inhibitory synapses. We also found that BPA administration reduces SYP and PSD95 expression in cortical pyramidal neurons. Previous studies have shown that SYP and PSD95 expression are decreased in some cortical areas in schizophrenia (Eastwood et al., 2000; Kristiansen et al., 2010). PSD95 is involved in glutamatergic transmission, synaptic plasticity and dendritic spine morphogenesis (Funke et al., 2005; Kim and Sheng, 2004). Our findings suggest that BPA exposure may decrease the activity of NMDA receptor-rich glutamatergic neurons in the cerebral cortex.

Dendritic spine density and morphology are affected in many neuropsychiatric disorders, particularly those that involve cognitive deficits. For example, some studies have reported that dendritic spine deficits may be more relevant for some cognitive symptoms, including schizophrenia and working memory deficits (Glausier and Lewis, 2013; Mahmmoud et al., 2015; Penzes et al., 2011). Moreover, the NMDA subtype of the glutamate receptor plays important roles in brain functions that have been implicated in schizophrenia (Komuro and Rakic, 1993). BPA exposure causes estrogen-associated changes relevant to schizophrenia (Brown, 2009). Moreover, BPA impairs memory function and glutamatergic homeostasis in mice (Jardim et al., 2017). We found that BPA administration in mice reduced locomotor activity and resulted in spatial memory deficit. These results thus suggest that BPA could modify neuronal circuitry, especially circuits involved in cognition, by modifying the postsynaptic compartment of excitatory synapses.

The pathogenic mechanisms underlying BPA-medicated neuroanatomical changes are not well understood. Herein, we demonstrate that BPA causes downregulation of BDNF/NTRK2 signaling via regulation of *Rgs4* transcription in cortical pyramidal neurons. We found that neurite formation- and function-related genes such as *Slco1c1*, *Sv2b*, *Cck*, *Kcnq3* and *Rgs4* are downregulated in BPA-exposed neurons. Moreover, we showed that BPA exposure downregulates both mRNA and protein levels of *Rgs4*. *Rgs4* regulates G protein signaling and might be a schizophrenia susceptibility gene; *Rgs4* deficit in prefrontal cortex contributes to the behaviors related to schizophrenia (Huang et al., 2018). Inhibition of RGS4 decreased BDNF expression, along with phosphorylation of NTRK2, which is the BDNF receptor (He et al., 2019) and has been reported to have a crucial role in neurite and dendritic spine architecture as well as synaptic plasticity (Gomes et al., 2006; Kellner et al., 2014; Kim et al., 2006). Consistently, we have shown that BPA exposure reduces BDNF and NTRK2 expression, and also inhibits phosphorylation of NTRK2. We have also shown that pharmacological inhibition of RGS4 downregulates expression of BDNF and NTRK2. Our findings suggest that BPA exposure may decrease RGS4 expression, resulting in reduced BDNF/NTRK2 signaling in cortical pyramidal neurons.

Many studies indicate that BDNF/NTRK2 signaling plays an essential role in synaptic formation and functions (Minichiello, 2009). BDNF induces an upregulation of SYP and Tau proteins via the activation of NTRK2 (Coffey et al., 1997). In addition, activation of BDNF increases PSD95 in dendritic spines (Hu et al., 2008). We found that BPA administration reduces SYP and PSD95 expression; however, pharmacological activation of NTRK2 rescues their expression. Our findings suggest that BPA exposure causes downregulation of synapse-related proteins such as SYP and PSD95 via inactivation of BDNF/NTRK2 signaling.

The pathological mechanisms underlying BPA-mediated cognitive symptoms are much more complicated. The current study mainly focuses on dysregulation of synaptic formation and function following BPA exposure. BPA dysregulates *Rsg4* transcription in cortical pyramidal neurons, leading to BPA-mediated abnormal synaptic formation, anxiety and mild cognitive dysfunction via regulation of BDNF/NTRK2 signaling.

## MATERIALS AND METHODS

### Reagents

The BPA used (CAS number 80-05-07, catalog number 239658, Sigma-Aldrich, St Louis, MO, USA) was more than 99% pure and dissolved in DMSO (CAS number 67-68-5, catalog number D8418, Sigma-Aldrich). CCG50014 (CAS number 883050-24-6, catalog number 4567) and 7,8-DHF (CAS number 38183-03-8, catalog number 3826) were from Tocris (Bristol, UK).

### Primary neuronal cultures

Primary neuronal culturing was performed as described previously (Ka et al., 2014a). In brief, cerebral cortices from E15 mice were isolated and dissociated with trituration after trypsin/EDTA treatment. Then, the cells were plated onto poly-D-lysine/laminin-coated coverslips and cultured in medium containing neurobasal medium, B27 and N2 supplements.

### Cell viability assay

Cell viability assay was performed as described previously (Lee et al., 2020). In brief, for analysis of cell viability, 1×10^4^ cells/well were seed in 96-well plates and incubated for 24 h at 37°C under humidified conditions (5% CO_2_ atmosphere). Then, cells were treated with BPA at concentrations of 0.01, 0.1, 1, 10, 100 and 1000 μM for 24 h. Tetrazolium salt from an EZ-Cytox Kit (WST-8 assay; DoGen, Seoul, South Korea) was added to each well at a final concentration of 0.5 mg/ml, and the cells were incubated for 2 h at 37°C under humidified conditions (5% CO_2_ atmosphere). Finally, absorbance was measured at 450 nm using a microplate reader (GloMax, Promega, Madison, WI, USA).

### Cell transfection

Neuronal transfection was performed as described previously (Ka et al., 2016a, 2014b). DNA constructs were transfected into attached cells using lipofectamine (Thermo Fisher Scientific, Waltham, MA, USA) according to the manufacturer's protocol.

### Animals and housing conditions

Mice (C57BL/6N) were purchased from Orient Bio Inc. (Seoul, South Korea). The animals were housed five mice per cage under temperature (23±3°C)- and humidity (30-70%)-controlled conditions with standard rodent chow and water available *ad libitum*, and were maintained on a 12 h light/dark cycle (lights on at 08:00). Mice were administered either BPA (50 µg/kg) or 0.1% DMSO (control) orally once a day for 2 months. All experimental procedures were approved by the Institutional Animal Care and Use Committee at the Korea Institute of Toxicology and met National Institutes of Health guidelines for the care and use of laboratory animals (KIT-IACUC; Approval Numbers 1910-0332 and 2008-0247).

### RT-PCR

RNA was extracted from cultured neurons using TRIZOL reagent (Thermo Fisher Scientific), and cDNA was synthesized from 1 μg of total RNA using oligo-dT and random hexamers using a Verso cDNA synthesis kit (Thermo Fisher Scientific). For quantitative analysis of mRNA expression, comparative real-time PCR was performed with the use of SYBR Green PCR Master Mix (Applied Biosystems) To perform the quantitative PCR run, a Step OnePlus real-time PCR system (Applied Biosystems) was used with the following cycle parameters: 95°C for 3 min, 35 cycles of 95°C for 5 s, and 60°C for 20 s. Quantitative PCR products were analyzed by StepOne Software v2.1 (Applied Biosystems). The primer sequences for the amplification of *Rgs4* were 5′-TTCCTGCGAACACAGTTCTT-3′ (forward) and 5′-GTCAATGTTCTCCTCGCTGTAT-3′ (reverse); and the primer sequences for the amplification of GAPDH were 5′-TGCACCACCAACTGCTTAGC-3′ (forward) and 5′-ATGCCAGTGAGCTTCCCGTT-3′ (reverse). Relative quantification of *Rgs4* mRNA expression with an internal control gene (*Gapdh*) was performed using the 2^−ΔΔCT^ method. All reactions were run in triplicate.

### Immunoblotting

Western blotting was performed as described previously (Ka and Kim, 2016; Ka et al., 2017a). Tissue lysates from the hippocampal region were prepared using RIPA buffer, and the sample was centrifuged at 20,960 ***g*** for 10 min at 4°C. The supernatant was collected, and protein content was determined using a Pierce BCA Protein Assay Kit (Thermo Fisher Scientific) following the manufacturer's protocol. Proteins were separated on 8%, 10% or 15% SDS-PAGE gradient gel and transferred onto PVDF transfer membrane (Thermo Fisher Scientific). Then, the membrane was incubated with rabbit anti-SYP (1:1000; ab32594, Abcam, Cambridge, UK), rabbit anti-PSD95 (1:1000; ab18258, Abcam), rabbit anti-BDNF (1:1000; ab108319, Abcam), mouse anti-RGS4 (1:500; sc-398348, Santa Cruz Biotechnology, Dallas, TX, USA) and mouse anti-β-actin (1:5000; A5316, Thermo Fisher Scientific) at 4°C overnight. Appropriate secondary antibodies conjugated to horseradish peroxidase were used (Thermo Fisher Scientific), and ECL reagents (Thermo Fisher Scientific) were used for immunodetection. For quantification of band intensity, blots from three independent experiments for each molecule of interest were used. Signals were measured using ImageJ software and represented as relative intensity versus control. β-actin was used as an internal control to normalize band intensity.

### Immunostaining

Immunostaining of brain sections or dissociated neural cells was performed as described previously (Ka et al., 2017b). The following primary antibodies were used: rat anti-KI67 (1:1000; 11-5698-82, Thermo Fisher Scientific), mouse anti-MAP2 (1:500; 13-1500, Thermo Fisher Scientific), rabbit anti-TUJ1 (1 μg/ml; ab18207, Abcam), mouse anti-VGLUT (1:1000; 135001, Synaptic Systems, Goettingen, Germany), mouse anti-VGAT (1:1000; 131011, Synaptic Systems), chicken anti-GFP (1:1000; A10262; Thermo Fisher Scientific), rabbit anti-GFP (1:1000; A11122; Thermo Fisher Scientific). Appropriate secondary antibodies conjugated with Alexa Fluor dyes (1:2000; Thermo Fisher Scientific) were used to detect primary antibodies.

### Golgi staining

Golgi staining was performed as described previously (Ka et al., 2016b). In brief, Golgi-Cox staining to obtain cortex spine density was conducted with an FD Rapid GolgiStain Kit (FD Neuro Technologies, Columbia, MD, USA) according to the manufacturer's instructions. Coronal tissue sections of 180 μm thickness were made at room temperature using a VT1000S (Leica Biosystems, Buffalo Grove, IL, USA). Sections were dehydrated with a gradient of 50%, 75%, 95% to 100% ethanol and cleared in xylene.

### Morphometry

Images of brain sections at periodic distances along the rostrocaudal axis were taken with a Zeiss LSM800 confocal microscope and ZEN software. Ten mice for each experiment (control mice, *n*=5; BPA-exposed mice, *n*=5) were used. Stereological analysis of immunostained cells was performed by analyzing one-in-six series of 40 μm coronal sections (240 μm apart). The images were subjected to software-driven particle analysis with automatic machine-set thresholding in ImageJ, thus eliminating subjective investigator bias. Then, a particle parameter enumeration analysis was followed for size exclusion at a minimum of 10 pixel^2^. The blue-channel images were used to assess background cells. For analyzing cultured cells, more than 20 fields scanned horizontally and vertically were examined in each condition. Cell numbers are described in figure legends. The calculated values were averaged, and some results were recalculated as relative changes versus control.

### Behavioral assays

All behavioral assays were done during light cycle. Behavior recording and analysis were performed by a researcher blinded to the genotype of each mouse. Health conditions including weight, activity and feeding were checked prior to assays.

#### Open field test

A mouse was placed near the wall side of a 20×20×π cm open field arena, and the movement of the mouse was recorded by a camera for 5 min. The recorded video file was further analyzed using EthoVision XT 7.0 software (Noldus, Wageningen, Netherlands). Total distance moved and average velocity of movement were recorded. The number of entries into, and the overall time spent in, the center of the arena (10×10×π cm imaginary circle) were also measured. The open field arena was cleaned with 70% ethanol between each trial.

#### Y-maze

A Y-maze task was used to assess spatial short-term memory, as described by Garcia and Esquivel (2018). The Y-maze was custom made from a wooden platform, which has three arms of equal length (35 cm), width (5 cm) and height (10 cm), connected at the center at an angle of 120°. As above, mice were divided into two groups to assess spontaneous alternations and working memory. The videos were recorded using a GoPro HERO7 Black Action Camera (GoPro Technology**,** San Mateo, CA, USA) and later analyzed with EthoVision XT 7.0 software (Noldus).

### RNA isolation and gene expression profiling

In the present study, we performed global gene expression analyses using Affymetrix arrays. Total RNA was isolated using Trizol reagent (Thermo Fisher Scientific). RNA quality was assessed by an Agilent 2100 Bioanalyzer (Agilent Technologies, Santa Clara, CA, USA), and quantity was determined by an ND-1000 spectrophotometer (NanoDrop Technologies, Wilmington, DE, USA). Per RNA sample, 300 ng was used as input into the Affymetrix procedure as recommended by protocol (https://www.affymetrix.com/support/downloads/manuals/expression_s2_manual.pdf). Briefly, 300 ng of total RNA from each sample was converted to double-stranded cDNA Using a random hexamer incorporating a T7 promoter, amplified RNA (cRNA) was generated from the double-stranded cDNA template though an *in vitro* transcription reaction and purified with the Affymetrix sample cleanup module. cDNA was regenerated through a random-primed reverse transcription using a dNTP mix containing dUTP. The cDNA was then fragmented by UDG and APE 1 restriction endonucleases and end labeled by terminal transferase reaction incorporating a biotinylated dideoxynucleotide. Fragmented end-labeled cDNA was hybridized to the Affymetrix arrays for 16 h at 45°C and 60 rpm as described in the Gene Chip Whole Transcript (WT) Sense Target Labeling Assay Manual (Affymetrix). After hybridization, the chips were stained using Streptavidin Phycoerythrin, washed in a Genechip Fluidics Station 450 (Affymetrix) and scanned using a Genechip Array scanner 3000 7G (Affymetrix).

### Data analysis

After the final wash and staining step, the Affymetrix array was scanned using an Affymetrix Model 3000 G7 scanner, and the image data were extracted through Affymetrix Command Console software version 1.1. The raw .cel file generated through the above procedure contained expression intensity data and was used for the next step. Expression data were generated by Affymetrix Expression Console software version 1.1. For the normalization, Robust Multi-Average algorithm implemented in Affymetrix Expression Console software was used.

### Electrophysiology

Whole-cell voltage-clamp recordings were obtained from cultured cortical pyramidal neurons. The external solution contained 150 mM NaCl, 3 mM KCl, 10 mM HEPES, 6 mM mannitol, 2.5 mM CaCl_2_ and 1.5 mM MgCl_2_, (pH adjusted to 7.40 with NaOH). Patch electrodes were made from borosilicate glass capillaries (Harvard Apparatus, Kent, UK) that were pulled to a tip resistance of 3-5 MΩ using a pipette puller (Narishige Scientific Instrument Lab., Tokyo, Japan) and filled with a solution containing 140 mM CsCl, 10 mM HEPES, 10 mM EGTA, 0.5 mM CaCl_2_, 1 mM Na_2_-ATP, 0.1 mM Na_2_-GTP and 10 mM QX314 (pH adjusted to 7.27 with CsOH). For recording mEPSCs, 1 μM tetrodotoxin and 100 μM picrotoxin were added to the external solution, and for recording mIPSCs 1 μM tetrodotoxin, 10 μM D-AP5 and 10 μM NBQX were added. The currents were recorded using Axopatch 200B (Molecular Devices, San Jose, CA, USA), and the neurons were held at a holding potential of −60 mV. The mEPSC and mIPSC recordings were analyzed using Mini Analysis software (Synaposoft, Atlanta, GA, USA).

### Statistical analysis

Normal distribution was tested using the Kolmogorov–Smirnov test, and variance was compared. Unless otherwise stated, statistical significance was determined by one-way or two-way analysis of variance (ANOVA) followed by the Bonferroni post hoc test for multiple comparisons. Data were analyzed using GraphPad Prism (GraphPad Software, La Jolla, CA, USA) and presented as mean±s.e.m. *P*-values are indicated in figure legends.

## Supplementary Material

Supplementary information
