# Supplementary material for: Bisphenol-A impairs synaptic formation and function by RGS4-mediated regulation of BDNF signaling in the cerebral cortex
Source: Dis Model Mech. 2022 Jul 25;15(7):dmm049177. doi: 10.1242/dmm.049177 (PMC9346518; doi:10.1242/dmm.049177)
Supplement: Supplementary information [file dmm-15-049177-s1.pdf]

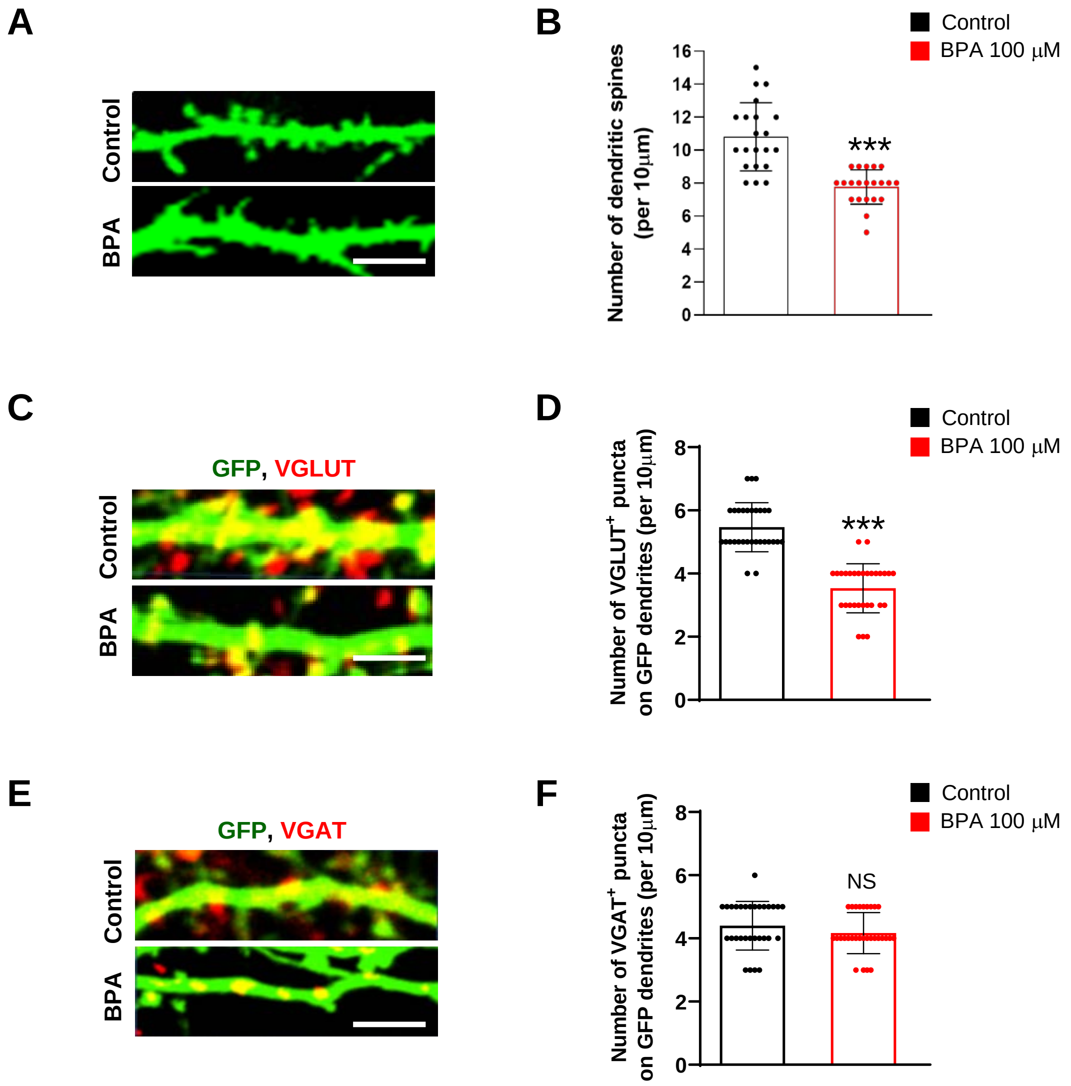

Fig. S1. BPA affects dendritic spine and synaptic formation in cultured hippocampal neurons

A BPA modifies dendrites spine density in cultured hippocampal neurons. Cultured hippocampal neurons were isolated from E15 embryos, cultured, and transfected with a GFP plasmid at DIV 10. After 4 days, neurons were treated with 100 μM BPA for 24 h. Scale bar, 5 μm. **B** Quantification of the number of dendritic spines in each condition. n=20 neurons from 3 independent cultures using 3 mice for each condition. Statistical significance was determined by one-way ANOVA with Bonferroni correction test. Data are shown as relative changes versus controls. ***p < 0.001. **C** Cultured hippocampal neurons from E15 mice were cultured for 10 days and transfected with a GFP plasmid. After 4 days, neurons were treated with 100 µM BPA for 24 h. Excitatory synapses were assessed by immunostaining using a VGLUT antibody. Scale bar, 5 µm. **D** Quantification of the number of excitatory synapses shown in a. n=30 neurons from 3 independent cultures using 3 mice for each condition. Statistical significance was determined by one-way ANOVA with Bonferroni correction test. Data are shown as relative changes versus controls. ***p < 0.001. **E** BPA induces no changes in the number of inhibitory synapses in cultured hippocampal neurons. Inhibitory synapses were assessed by immunostaining using a VGAT antibody. Scale bar, 5 µm. **F** Quantification of inhibitory synapse numbers shown in c. n=30 neurons from 3 independent cultures using 3 mice for each condition. Statistical significance was determined by one-way ANOVA with Bonferroni correction test. Data are shown as relative changes versus controls.

Disease Models & Mechanisms • Supplementary information

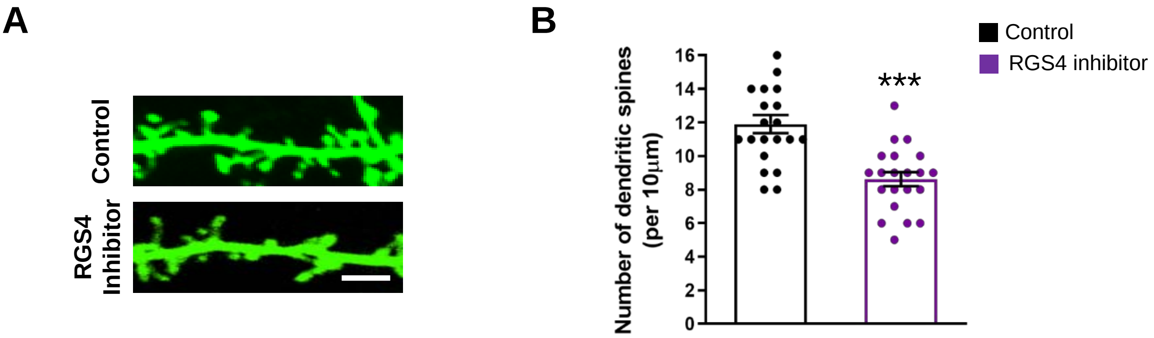

Fig. S2. CCG50014 (RGS4 inhibitor) affects dendritic spine and synaptic formation in cultured cortical neurons

A CCG50014 modifies dendrites spine density in cultured cortical neurons. Cultured cortical neurons were isolated from E15 embryos, cultured, and transfected with a GFP plasmid at DIV 10. After 4 days, neurons were treated with 10 μM CCG50014 for 24 h. Scale bar, 5 μm. **B** Quantification of the number of dendritic spines in each condition. n=20 neurons from 3 independent cultures using 3 mice. Statistical significance was determined by one-way ANOVA with Bonferroni correction test. Data are shown as relative changes versus controls. ***$p < 0.001$.

Disease Models & Mechanisms • Supplementary information
